# Association between polymorphisms in the flanking region of the *TAFI* gene and atherosclerotic cerebral infarction in a Chinese population

**DOI:** 10.1186/1476-511X-13-80

**Published:** 2014-05-13

**Authors:** You Li, Zhiliang Zeng, Jianghao Zhao, Guoda Ma, Lili Cui, Hua Tao, Zhijun Lin, Yanyan Chen, Bin Zhao, Yusen Chen, Keshen Li

**Affiliations:** 1 Guangdong Key Laboratory of Age-Related Cardiac and Cerebral Diseases, Affiliated Hospital of Guangdong Medical College, Zhanjiang 524001, China; 2 Department of Neurology, Affiliated Hospital of Guangdong Medical College, Zhanjiang 524001, China

**Keywords:** Thrombin activatable fibrinolysis inhibitor (TAFI), Atherosclerotic Cerebral Infarction (ACI), Polymorphism

## Abstract

**Background:**

Atherosclerosis is the leading etiologic factor of Atherosclerotic Cerebral Infarction (ACI). Previous studies have shown that thrombin activatable fibrinolysis inhibitor (TAFI) may play an important role in the occurrence of acute cerebral infarction, and the levels of TAFI are affected by several single nucleotide polymorphisms (SNPs) located in the regulatory and coding regions of the gene encoding TAFI. The present study aimed to determine whether polymorphisms (*TAFI –*2345 2G/1G, –1690 A/G, –438 A/G, +1583 A/T) of the *TAFI* gene were associated with ACI in a Han Chinese population.

**Methods:**

The variant genotypes were identified by restriction fragment length polymorphism (RFLP) and allele-specific polymerase chain reactions (AS-PCR) in 225 patients with ACI and 184 age-matched healthy individuals.

**Results:**

There was a significant difference in the genotype and allele frequencies of *TAFI –*2345 2G/1G and −1690 A/G polymorphisms between the ACI and control subjects. Further stratification analysis by gender revealed that the presence of the –438 AA genotype and the A allele conferred a higher risk of developing ACI in male patients (p < 0.05). Haplotype analysis demonstrated that four haplotypes of *TAFI* are significantly associated with ACI.

**Conclusions:**

Our study provides preliminary evidence that the *TAFI –*2345 2G/1G and –1690 A/G polymorphisms are associated with ACI susceptibility in a Han Chinese population.

## Background

Thrombin activatable fibrinolysis inhibitor (TAFI), also known as carboxypeptidase B2 (CPB2), is a basic carboxypeptidase [1,2]. TAFI can be activated by trypsin-like enzymes, such as thrombin, plasmin, and the thrombin/thrombomodulin complex. Activated TAFI removes C-terminal lysine residues on plasmin-modified, partially degraded fibrin and attenuates the rate of plasminogen activation, thus playing a significant role in blood coagulation and fibrinolysis. An imbalance between the processes of coagulation and fibrinolysis is one of the main contributors to thrombosis, a key factor for the onset of atherosclerotic cerebral infarction (ACI) [1,3-5]. Impaired fibrinolysis may also be a risk factor for arterial thrombotic events [6]. In addition, an increased plasma TAFI concentration may be a risk factor for multiple diseases, including stable angina pectoris [7], coronary artery disease [8,9], myocardial infarction (MI) [10], and venous thrombosis [11-13]. Therefore, the risk of cardiovascular disease, in general, may be decreased by lowering plasma TAFI levels.

The *TAFI* gene maps to chromosome 13q14.11, spans approximately 48 kb and contains 11 exons. Several SNPs located in the flanking region of *TAFI* have been shown to modify plasma TAFI antigen levels [14-16]. Franco *et al.* demonstrated that polymorphisms in the *TAFI* promoter determine plasma antigen levels and may influence the risk of venous thrombophilia [14]. Henry *et al.* revealed that circulating levels of TAFI are strongly determined by polymorphic variations in the promoter and the 3'-untranslated region (UTR) of the *TAFI* gene [15]. In addition, Biswas *et al.* determined that TAFI antigen levels were significantly associated with the disease phenotype and with *TAFI* polymorphisms [17]. These findings suggest that *TAFI* polymorphisms may play a role in modulating plasma TAFI antigen level, and thus contribute to ACI phenotype.

Association studies between polymorphisms of *TAFI* and stroke have been reported for many different origin populations, but with conflicting results [17-19]. Akatsu *et al.* found no statistical correlation between *TAFI* Ala147Thr and Thr325Ile polymorphisms and cerebral infarction [18]. Ladenvall *et al.* also did not find any association between genetic variants of *TAFI* and overall ischemic stroke, but they did find an increased risk between *TAFI* polymorphisms and stroke subtypes [20]. More recently, Kozian *et al.* found that the Ile/Ile genotype at position 325 of *TAFI* is associated with the incidence of stroke and the age at onset of first stroke [19]. Furthermore, the incidence of stroke is higher in *TAFI* Ile325Ile patients with predisposing risk factors for thrombotic events, such as diabetes mellitus, myocardial infarction or hypertension [19]. These lines of evidence suggest a role for *TAFI* polymorphisms in the pathogenesis of stroke. However, the association between *TAFI* polymorphisms and ACI has not yet been determined. Therefore, the present study aims to evaluate the existence of an association between four polymorphisms (−2345 2G/1G, –1690 A/G, –438 A/G and +1583 A/T) of *TAFI* and ACI in a case-controlled study of a Han Chinese population.

## Methods

### Study population

The study recruited 225 patients with ACI (145 males and 80 females) from the Department of Neurology at the Affiliated Hospital of Guangdong Medical College in China between 2011 and 2013. Patients’ diagnoses were verified with either computed tomography (CT) or magnetic resonance imaging (MRI) and were clinico-neuropathologically confirmed. Patients were classified into subtypes according to the Trial of Org 10172 in Acute Stroke Treatment (TOAST) classification [21]. Patients with transient ischemic attacks, cardiogenic cerebral infarctions, cerebral hemorrhage, coronary artery diseases, autoimmune diseases, systemic inflammatory diseases, blood diseases, or malignant tumors were excluded from this study. Additional information recorded included age, gender, cerebrovascular disease risk factors and previous and ongoing medication or systemic therapy.

A total of 184 matched controls (107 males and 77 females) were recruited from the Health Examination Center of the Affiliated Hospital of Guangdong Medical College during the same time period. These control patients were comparable to the ACI subjects in age, sex, and race. The same exclusion criteria were used as above. Written informed consent was obtained from each participant prior to enrollment in the study. The study was approved by the ethics committee of Guangdong Medical College and was conducted according to the principles of the Declaration of Helsinki.

### Genotyping of *TAFI* polymorphisms

Genomic DNA was extracted from peripheral blood using the EZ-10 Spin Column Whole Blood Genomic DNA Isolation Kit (Sangon Biotech®, Shanghai, China) according to the manufacturer’s instructions. Four *TAFI* SNPs (−2345 2G/1G, –1690 A/G, –438 A/G and +1583 A/T) were selected according to previous findings [15]. Genotyping of the *TAFI* gene for the presence of –438 A/G and +1583 A/T mutations was performed by polymerase chain reaction (PCR) and restriction fragment length polymorphism (RFLP); *TAFI –*2345 2G/1G and –1690 A/G mutations were identified by allele-specific polymerase chain reaction (AS-PCR). The primers used are listed in Table 1. The PCR and RFLP analysis were performed as described previously [22]. For quality control, we sequenced 5% DNA samples directly for each SNP, and no inconsistencies were detected. The results were read and interpreted in a blind fashion, without knowledge of the patient’s designated group.

**Table 1:** Methods and primers used in genotyping of TAFI −438 A/G, +1583 A/T, −1690 A/G and −2345 2G/1G polymorphisms

**Gene polymorphisms**	**Analysis**	**Primers**
TAFI −438 A/G	PCR-RFLP	Forward:ACCAATGGGAATGTAGG
		Reverse:CCTTGCCTCTGACTTTT
TAFI +1583 A/T	PCR-RFLP	Forward:CTTGGCATGTCATTAGG
		Reverse:TGCGGCATTTGTTGACA
TAFI −1690 A/G	AS-PCR	Common Forward a:CACCTGTAGACTTTTGC
		Special Forward a1:TTAACTATTTTGACTGTTTA
		Special Forward a2:TTAACTATTTTGACTGTTTG
		Common Reverse b: CACTGAAGGAGAGAAAG
TAFI −2345 2G/1G	AS-PCR	Common Forward a:CACCTCAACTGGACTATGT
		Special Forward a1:AGTTTTTAAAACATGAAAGA
		Special Forward a2:AGTTTTTAAAACATGAAAGGA
		Common Reverse b: TGTTCCCTTGCAGTTTAGC

### Statistical analysis

All statistical tests were performed using SPSS 19.0 software (IBM, Armonk, NY, USA). The Hardy-Weinberg equilibrium and differences in the gene and allele frequencies between groups were assessed using a *χ*^2^ test. The allele and genotype frequency differences between patient cases and healthy controls were assessed by Fisher's exact test or the chi-square test. For comparisons resulting in significant statistical values, an odds ratio (OR) with a 95% confidence interval (95% CI) was calculated by comparing the total number of each allele found among the cases and controls. Haplotype analyses were conducted by SHEsis software (http://analysis.bio-x.cn/myAnalysis.php) [23]. All p values reported were two-tailed. Statistical significance was defined as p < 0.05.

## Results

### Baseline characteristics

The clinical characteristics of the 409 participants (225 ACI patients and 184 control subjects) are presented in Table 2. Conventional risk factors, including smoking, hypertension, and diabetes, were significantly more common in the ACI group than in the control group. There were no significant differences in age or sex between the ACI patients and controls. The mean ages of the ACI and control patients were 67.60 years old (±10.61 years) and 66.11 years old (±11.02 years), respectively. In ACI patients, the blood glucose levels tended to be higher than in the controls, but the high-density lipoprotein (HDL) levels were lower at admission. The cholesterol, triglyceride and low-density lipoprotein (LDL) levels of the ACI patients were not significantly different from those of the healthy control patients.

**Table 2:** General characteristics of patients with ACI and controls

**Variables**	**Cases (n = 225)**	**Controls (n = 184)**
Age (years) (mean ± SD)	67.60 ± 10.61	66.11 ± 11.02
Sex (male/female)	145/80	107/77
Smoking (%, n)	21.8%(49) *	13.0%(24)
Hypertension	64.4%(145) *	23.9%(44)
Diabetes	23.1%(52)*	6.0%(11)
Blood glucose levels (mmol/L)	6.51 ± 2.60*	5.43 ± 1.70
Cholesterol (mmol/L)	5.17 ± 1.35	5.30 ± 1.18
Triglyeride (mmol/L)	1.23 ± 0.72	1.27 ± 0.86
High density lipoprotein (HDL)(mmol/L)	1.28 ± 0.28*	1.45 ± 0.41
Low density lipoprotein (LDL) (mmol/L)	3.36 ± 1.02	3.31 ± 0.94

*P < 0.05.

### Genotype and allele frequencies of *TAFI* polymorphisms in ACI patients and controls

The genotype and allele frequencies of the *TAFI* gene polymorphisms are shown in Table 3. No deviation from the Hardy-Weinberg equilibrium for the polymorphisms examined was observed in the genotype distributions of the ACI patients and controls (data not shown).

**Table 3:** **Allele and genotype frequencies of the *TAFI *** **polymorphisms in ACI patients and controls**

**TAFI Polymorphisms**	**Frequency**	
	**Cases (n = 225)**	**Controls (n = 184)**
−2345 2G/1G		
2G/2G	66(29.3%)	42(22.8%)
2G/1G	125(55.6%)	97(52.7%)
1G/1G	34(15.1%)*	45(24.5%)
2G	257(57.1%)	181(49.2%)
1G	193(42.9%)*	187(50.8%)
−1690 A/G		
AA	110(48.9%)*	72(39.1%)
AG	96(42.7%)	86(46.7%)
GG	19(8.4%)	26(14.2%)
A	316(70.2%)*	230(62.5%)
G	134(29.8%)	138(37.5%)
−438A/G		
GG	122(54.2%)	108(58.7%)
GA	87(38.7%)	67(36.4%)
AA	16(7.1%)	9(4.9%)
G	331(73.6%)	283(77.0%)
A	119(26.4%)	85(23.0%)
+1583 A/T		
TT	129(57.3%)	99(53.8%)
TA	81(36.0%)	78(42.4%)
AA	15(6.7%)	7(3.8%)
T	339(75.3%)	276(75.0%)
A	111(24.7%)	92(25.0%)

*P < 0.05.

The comparison of genotype distributions between the ACI subjects and control subjects revealed that there was a statistically significant association between the –2345 2G/1G and –1690 A/G polymorphisms of *TAFI* and the risk of ACI. The 2G versus 1G allele frequencies of the *TAFI –*2345 2G/1G polymorphism were 57.1% versus 42.9% among the patient cohort and 49.2% versus 50.8% among control subjects (p < 0.05). The prevalence of the *TAFI –*2345 2G/2G (p = 0.017, OR = 0.550, 95% CI: 0.335-0.903) genotype and the −2345 2G (p = 0.024, OR = 0.727, 95% CI: 0.551-0.959) allele frequencies was significantly higher in patients than in controls. Likewise, the A versus G allele frequencies of the *TAFI –*1690 A/G polymorphism were 70.2% versus 29.8% among the patient cohort and 62.5% versus 37.5% among control subjects (p < 0.05). The prevalence of the –1690 AA genotype (p = 0.048; OR = 1.488, 95% CI: 1.002-2.209) and the –1690 A allele (p = 0.048; OR = 1.488, 95% CI: 1.002-2.209) frequencies was significantly higher in patients than in controls. No differences in the –438 A/G and +1583 A/T genotype and allele frequencies were observed between ACI cases and control subjects (Table 3).

### Stratification of the genotype and allele frequencies of *TAFI* polymorphisms in ACI patients and controls by gender

When stratified by gender, a significant difference was found in the frequency of the A allele of the *TAFI –*438 A/G polymorphism between male ACI patients and male controls (p = 0.039; OR = 1.549 (95% CI: 1.020-2.353). In female individuals, no significant difference of genotype or allelic distribution was found between the ACI and control groups. We did not observe differences of *TAFI* +1583 A/T, –2345 2G/1G and –1690 A/G genotype and allele frequencies between ACI patients and controls when separated by gender (Table 4).

**Table 4:** Subgroup analysis of TAFI -438A/G polymorphism genotype and allele frequencies of the men and women

**Gender**	**Group**	**Number**	**GG**	**GA**	**AA**	**G**	**A**
Male	Cases	145	75(51.7%)	57(39.3%)	13(9.0%)*	207(71.4%)	83(28.6%)*
	Controls	107	65(60.7%)	40(37.4%)	2(1.9%)	170(79.4%)	44(20.6%)
Female	Cases	80	47(58.8%)	30(37.4%)	3(3.8%)	124(77.5%)	36(22.5%)
	Controls	77	43(55.8%)	27(35.1%)	7(9.1%)	113(73.4%)	41(26.6%)

*Compare with control group, *P* < 0.05.

### Haplotype analysis

We found a total of 16 haplotypes. Four haplotypes in which the frequency of the haplotype was greater than 5% in ACI cases and controls were included in the haplotype analysis. The haplotypes included yielded strongly significant associations (Table 5). The frequency of haplotypes H2 (2G/A/G/A) and H3 (2G/A/G/T) was significantly higher in the ACI case group than in the control group (p = 0.026; p = 0.002, respectively); whereas the haplotype frequencies for H1 (1G/G/G/T) and H4 (2G/G/G/T) were lower in the ACI cases (p = 0.015 and p = 0.001, respectively).

**Table 5:** Association analysis of TAFI haplotypes

**NO**	**Haplotypes**				**Cases**	**Controls**	***P***	**OR**	**95% CI**
	**−2345**	**−1690**	**−438**	**+1583**					
H1	1G	G	G	T	22.71(5.0%)	34.50(9.4%)	0.015	0.512	0.296 ~ 0.887
H2	2G	A	G	A	25.50(5.7%)	9.26(2.5%)	0.026	2.328	1.083 ~ 5.005
H3	2G	A	G	T	131.72(29.3%)	72.87(19.8%)	0.002	1.684	1.212 ~ 2.340
H4	2G	G	G	T	19.64(4.4%)	37.62(10.2%)	0.001	0.399	0.227 ~ 0.703

Only haplotypes whose frequency was >5% in both cases and controls were included in the Table.

## Discussion

The results of the present study provide evidence that the variant –2345 2G/1G and –1690 A/G polymorphisms of *TAFI* are associated with the risk of developing ACI in contrast with the –438 A/G and +1583 A/T polymorphisms. To the best of our knowledge, this result is the first to suggest an association of the –2345 2G/1G and –1690 A/G polymorphisms of *TAFI* with ACI in a Chinese population. Further stratification by gender revealed that the –438 A/G polymorphism of *TAFI* is associated with ACI in male patients. In addition, haplotype analysis demonstrated that four haplotypes are significantly associated with ACI.

TAFI is a 58-kDa plasma glycoprotein secreted by hepatocytes as an inactive form [24]. Upon activation by thrombin, plasmin, or the thrombin-thrombomodulin complex, TAFI is transformed into a carboxypeptidase B-like enzyme (TAFIa) [2,25]. TAFIa plays an important role in regulating the balance between coagulation and fibrinolysis by inhibiting fibrinolysis. Previous studies have indicated that high levels of TAFI in circulating plasma increase the risk of cardiovascular death during the acute phase of ischemic stroke [24]. In addition, recent studies have reported that TAFI deficient mice are also susceptible to intracerebral thrombosis and ischemic stroke [26]. These lines of evidence lead to the hypothesis that TAFI is of pathogenic significance to ACI.

Several studies have analyzed the relationship between *TAFI* polymorphisms and cardiovascular disease, with different and even contradictory conclusions drawn from various ethnical populations. While one study found that the 147Thr allele of the Ala147Thr polymorphism protected against MI [27], in another study, the 147Thr allele was associated with a higher risk of angina [28]. *TAFI* polymorphisms C+1542G and Thr325Ile were also found to be related to the type of ACS [29] and thrombotic microangiopathies [30]. Regarding cerebrovascular diseases, Akatsu *et al.* did not find any association between the Ala147Thr and Thr325Ile polymorphisms of *TAFI* and cerebral infarction [18]. However, a prospective cohort study in Europe demonstrated that the Thr325Ile polymorphism of *TAFI* is associated with the incidence of stroke and the age at onset of first stroke in patients [19]. Ladenvall *et al.* did not find any association between a series of tagging SNPs of the *TAFI* gene, but an increased risk was found between *TAFI* polymorphisms and stroke subtypes [20]. In the present study, we show, for the first time, that the −2345 2G/1G and −1690 A/G polymorphisms of *TAFI* were associated with ACI in a Chinese population. As demonstrated by Henry *et al.*, the four polymorphisms investigated in the present study were in strong linkage disequilibrium with each other and with the previously described Ala147Thr polymorphism [15]. It is most likely that these polymorphisms function synergistically to contribute to the disease phenotype.

It has been widely recognized that plasma TAFI levels are strongly genetically controlled [17,31]. TAFI levels are under the control of several SNPs located in the regulatory and coding regions of the *TAFI* gene [17,18,25,32]. A transethnic study using fine mapping of quantitative trait nucleotides (QTNs) underlying TAFI antigen levels identified that the −2345 2G/1G SNP located in the 5' region might be one of the QTNs [33] and that −2345 1G alleles were independently associated with increased TAFI levels. The −1690 A/G polymorphism is located two bases downstream from a potential binding site for the HFH3 transcription factor, and the −438 A/G polymorphism is located one base downstream from a potential binding site for the VBP transcription factor [14]. These polymorphisms may alter the binding of the corresponding transcription factors to the promoter, thus affecting promoter activity and contributing to varied TAFI expression. In the 3’-UTR, there are three transcription termination sites [34]. The interaction of cis-acting elements within the 3'-UTR of *TAFI* with IL-1 and IL-6 significantly increased the stability and frequency of mRNA transcripts [35]. In addition, polymorphisms in the 3’-UTR may alter TAFI mRNA abundance by influencing mRNA processing or stability. The haplotype carrying the +1583 T allele reduces the stability of *TAFI* transcripts, suggesting the +1583 A/T allele is also related to TAFI antigen levels [14,15]. Data from Africans and Europeans converged to identify the 1583 T/A polymorphism as one of the QTNs contributing to elevated TAFI levels. In sum, polymorphisms both in the promoter and 3’-UTR regions of *TAFI* can affect the binding of transcription factors or mRNA stability, thereby influencing gene expression.

The stratified analysis by gender revealed that among males, those with the variant −438 A allele and AA genotype had a significantly higher risk of ACI, while among women, no statistical significance was found. TAFI levels in patients with polycystic ovary syndrome (PCOS) were significantly higher than in control groups [36,37]. It is known that PCOS patients express high levels of androgen [36,37]. The androgen may affect TAFI production by some unknown mechanism. Therefore, it is conceivable that the −438 AA allele that is associated with higher androgen levels could also increase TAFI levels in male ACI patients. However, more functional studies on the relationship between sex hormones and TAFI levels should be carried out.

In genes with multiple susceptibility alleles, particularly when the LD between polymorphisms is weak, haplotype-based association studies have advantages over analyses based on individual polymorphisms [38]. In the present study, haplotype analysis identified four haplotypes significantly associated with ACI. H1(1G/G/G/T) and H4(2G/G/G/T) were associated as protective haplotypes, while H2(2G/A/G/A) and H3(2G/A/G/T) were risk haplotypes. The haplotype carrying the G allele of −1690 is a protective haplotype, while the A allele of −1690 is a risk haplotype. Ladenvall *et al.* found that *TAFI* genotypes and haplotypes showed significant associations with TAFI levels. While no haplotype was found to have a significant association with overall ischemic stroke, subtype analysis revealed an association between the H2B haplotype and cryptogenic stroke and an association between the H1B haplotype and small-vessel disease [20]. As these results illustrate, haplotype analysis is helpful for deciphering the relationship between multiple SNPs and disease susceptibility. Our findings suggest that the haplotype of *TAFI* could be genetic a marker for ACI.

There are several limitations to this study. The small number of participants in this study was insufficient and therefore may have led to non-representative results. This limitation may have suppressed a true relationship due to a type II statistical error. A sample size with sufficient statistical power is critical to analyze the genetic associations of causal genes with complex diseases susceptibility. The sample size for detecting associations between disease and SNP markers is known to be highly affected by disease prevalence, disease allele frequency, linkage disequilibrium, inheritance models (e.g., additive, dominant, and multiplicative models), and effect size of the genetic variants (e.g., odds ratio, relative risk, etc.). Particular attention must be paid to sample sizes when more than one variable is studied simultaneously, such as multiple imperfectly correlated traits, intergenic interactions or gene-environment interactions [39]. Additionally, the comparatively small sample size, together with other environmental risks may mask the genuine differences in allele frequencies between cases and controls. Hong *et al.* suggested that a lower sample size for testing more common SNPs with stronger effect sizes and increased LD between marker allele and disease allele might contribute to achieve adequate statistical power [40]. Therefore, our results should be interpreted with caution. In addition, selection bias in the patient or control populations cannot be entirely excluded. The other clinical characteristics of the study group, such as hypertension, diabetes or hypercholesterolemia, might have complicated the associations between *TAFI* polymorphisms and ACI. Other functional polymorphisms might also influence TAFI expression, and their combined effects must be studied to improve the prediction of the occurrence, severity, and outcomes of ACI. Furthermore, the levels of TAFI were not evaluated. However, the primary purpose of our study was to establish a genetic reference for future studies. Thus, this investigation focused on assessing the association of different genetic polymorphisms of *TAFI* with the risk of developing ACI. Larger patient and control cohorts will be needed to confirm the association of *TAFI* gene polymorphisms with ACI in other populations.

## Conclusion

Our study is the first to describe the existence of an association between the −2345 2G/1G and −1690 A/G polymorphisms of *TAFI* and the risk of developing ACI in a Chinese population. Our findings support the idea that polymorphisms of *TAFI* contribute to the development of ACI. Our study may provide clues for the evaluation of individual susceptibility to ACI as well as for effective measures for the control and prevention of ACI. More work is required to shed light on the role of TAFI in the pathogenesis of ACI and to further clarify its prognostic and therapeutic potential.

## Abbreviations

TAFI: Thrombin activatable fibrinolysis inhibitor; ACI: Atherosclerotic cerebral infarction; SNP: Single nucleotide polymorphism; RFLP: Restriction fragment length polymorphism; AS-PCR: Allele-specific polymerase chain reaction; TOAST: Trial of Org 10172 in Acute Stroke Treatment; OR: Odds ratio; CI: Confidence interval; QTNs: quantitative trait nucleotides.

## Competing interest

The authors have no actual or potential conflicts of interest related to this manuscript. Appropriate approval was obtained, and the appropriate procedures were followed concerning human subjects.

## Authors’ contributions

LY, ZZL and ZJH carried out the molecular studies and drafted the manuscript. MGD, CLL and TH carried out the genotyping. CYY and LZJ performed the statistical analysis. CYS and LKS conceived of the study, and participated in its design and coordination. ZB helped to draft the manuscript. All authors read and approved the final manuscript.
